# Influenza Virus Surveillance from the 1918 Influenza Pandemic to the 2020 Coronavirus Pandemic in New York State, USA

**DOI:** 10.3390/v16121952

**Published:** 2024-12-20

**Authors:** Kay L. Escuyer, Donna L. Gowie, Kirsten St. George

**Affiliations:** 1 Wadsworth Center, David Axelrod Institute, New York State Department of Health, Albany, NY 12208, USA; kirsten.st.george@health.ny.gov; 2 Bureau of Communicable Disease Control, Empire State Plaza, New York State Department of Health, Albany, NY 12237, USA; donna.gowie@health.ny.gov

**Keywords:** influenza virus, surveillance, pandemics, preparedness, disease outbreaks, public health, sample size, database management systems

## Abstract

A historical perspective of more than one hundred years of influenza surveillance in New York State demonstrates the progression from anecdotes and case counts to next-generation sequencing and electronic database management, greatly improving pandemic preparedness and response. Here, we determined if influenza virologic surveillance at the New York State public health laboratory (NYS PHL) tests sufficient specimen numbers within preferred confidence limits to assess situational awareness and detect novel viruses that pose a pandemic risk. To this end, we analyzed retrospective electronic data on laboratory test results for the influenza seasons 1997–1998 to 2021–2022 according to sample sizes recommended in the Influenza Virologic Surveillance Right Size Roadmap issued by the Association of Public Health Laboratories and Centers for Disease Control and Prevention. Although data solely from specimens submitted to the NYS PHL were insufficient to meet surveillance goals, when supplemented with testing data from clinical laboratories participating in surveillance programs, the recommended surveillance goals were achieved. Despite the sudden decline in influenza cases in 2020–2021, impacted by the COVID-19 mitigation measures, the dramatic increases in influenza cases surrounding the coronavirus pandemic reveal that influenza remains a national and international public health threat. Sample submissions to public health laboratories must be encouraged to facilitate monitoring for emerging viruses and preparedness for another pandemic.

## 1. Introduction

The most significant disease threat of the 20th century arrived on 14th August 1918, in the New York City harbor on a Norwegian steamship carrying eight passengers who were ill with a disease known as “Spanish” influenza [1]. Maritime records indicate approximately 180 persons affected with influenza arrived on ships bound for New York City between July and September 1918 [1]. The extent of the subsequent epidemic was measured through vital statistics, military records, and newspaper articles. Those aged 15 to 45 suffered the greatest mortality [2], an unusual feature of the 1918 pandemic. An estimated 50 million deaths occurred worldwide [3], with 650,000 deaths in the USA, including 26,502 deaths recorded in New York State (NYS), notably higher than the number recorded for any subsequent influenza pandemic. After the discovery of influenza viruses, the 1918 pandemic was determined to have been caused by the influenza virus subtype designated A(H1N1) [4].

The next pandemic in 1957, caused by the “Asian” influenza A(H2N2) virus, was responsible for an estimated 600 deaths in New York State [5,6], 116,000 deaths nationwide [7], and 1.1 million deaths worldwide [8], with the over-65 age group most severely affected [9]. The subsequent 1968 Hong Kong influenza A(H3N2) pandemic caused an estimated 800 deaths in NYS [10,11] and 100,000 USA deaths [7], with less age-specific susceptibility [12,13]. Vital statistics attributed fatalities to influenza only when influenza was listed as the underlying cause of death. This is an underestimate of the true number of deaths due to influenza. School and workplace absenteeism reported to the health department provided a measure of influenza activity [14].

In 2009, while the world anticipated an H5N1 influenza pandemic from birds in Asia, a novel H1N1 strain emerged in pigs in Mexico and subsequently spread throughout the susceptible global human population. Initially in the US, only the US Centers for Disease Control and Prevention (CDC) Human Influenza Real-Time reverse transcription PCR Diagnostic (CDC Flu rRT-PCR Dx) Panel, available at US public health laboratories (PHLs), differentiated this strain from other seasonal A(H1N1) influenza viruses [15,16]. Later, in 2009, clinical and commercial laboratories implemented additional molecular tests for this new pandemic virus, expanding available laboratory diagnostics. Subsequently known as influenza A(H1N1)pdm09, the virus quickly circulated throughout the Americas and the world. The NYSDOH (NYS Department of Health) Bureau of Communicable Disease Control (BCDC) database shows 53,930 laboratory-confirmed influenza cases for 2009 [17], with children aged 5–14, the most affected [18]. Testing at the NYS PHL, renamed the Wadsworth Center in 1984, identified the majority of these cases as being caused by the pandemic influenza A(H1N1)pdm09 strain. NYS vital statistics in 2009 attributed 50 total deaths to influenza [19], whereas the National Center for Health Statistics for 2009 estimated 12,469 total USA deaths [20], and modeling of WHO FluNet data estimated between 123,000 and 203,000 worldwide pandemic influenza respiratory deaths [21].

The 2009 influenza pandemic confronted healthcare and surveillance systems already impacted by the economic recession. In 2010, the CDC and the Association of Public Health Laboratories (APHL) began a three-year collaborative effort with stakeholders to discern the complexities of the existing national virologic surveillance systems for influenza and assess the capabilities to optimize this surveillance. This culminated in the development of the Influenza Virologic Surveillance Right Size Roadmap [22]. Introduced in 2013 and updated in 2022, this “Right Size Roadmap” provides a guide to help prioritize public health influenza surveillance measures; standardizes strategies to best utilize limited resources; enhances the precision of influenza surveillance systems for monitoring activity and severity of the disease; and with prompt detection of an emerging virus enables an effective public health response to mitigate a potential pandemic. Statistical tools were developed as online calculators to determine the minimum number of specimens needed for testing by each state PHL. These minimum thresholds are determined primarily by the size of the patient population being monitored, with adjustments for disease prevalence when calculating for novel detection. The sample size goal for assessing situational awareness in NYS is to test at least 137 samples per week. To detect a novel virus in NYS with recommended statistical power, the sample size to test should be at least 124 samples per week during the high season and 36 samples per week during the low or shoulder season of influenza activity.

**Figure 1:**
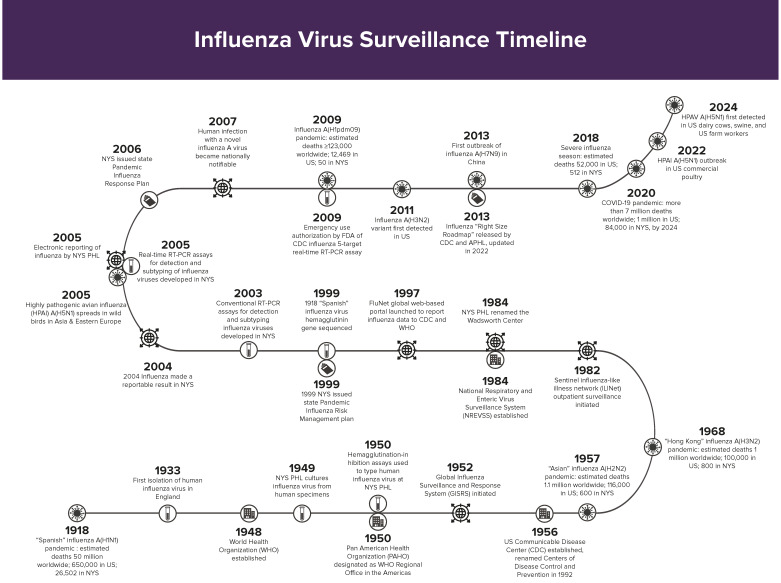
Timeline of influenza virus surveillance indicating global, US national, and New York State events from 1918 to 2024.

The hundred-year anniversary of the 1918 pandemic coincided with a particularly severe 2017–2018 influenza season in the USA, with an estimated 52,000 deaths, 83% of which occurred in older adults [23], testing the capacity of current surveillance systems and challenging available medical resources. This was soon overshadowed by the 2020 novel coronavirus pandemic that killed over 7 million people worldwide [24], 1 million in the USA [25], and 84,000 in NYS [26] by 2024 (Figure 1).

Historically, since the establishment of public health laboratories in the late nineteenth century, the spread of influenza disease was estimated through vital statistics [4,27]. The tremendous progress subsequently achieved through establishing influenza surveillance networks and diagnostic tests has enabled this detailed analysis of electronic NYS influenza data, including the impact of the coronavirus pandemic. We analyze twenty-five years of electronic laboratory data from 1997 to 2022 retrospectively with the Right Size Roadmap sample size goals to ascertain the progress in NYS during that time in achieving statistically significant data for the assessment of influenza viral disease.

## 2. Methods

### 2.1. Progression of Influenza Surveillance for Situational Awareness

The 1918 influenza pandemic highlighted the necessity for improved communication regarding disease threats to public health. After two world wars, political reorganization led to the establishment of the World Health Organization (WHO) in 1948 and the designation of the Pan American Health Organization (PAHO, originally established in the 19th century [28]) as the WHO Regional Office in the Americas in 1950. Soon thereafter, the Global Influenza Surveillance Network was formed in 1952, and the US Communicable Disease Center (CDC) as a WHO Collaborating Center in 1956 [29]. A national sentinel program for influenza began in 1982, with annual grant funding from the CDC aimed at enlisting physicians in each state to participate in surveillance of outpatient influenza-like illness (ILI). To contribute to this outpatient ILI surveillance network, known as the ILINet, respiratory specimens collected at enrolled physicians’ offices are sent to PHLs for testing. To monitor the temporal and geographic spread of influenza and respiratory syncytial virus, participating U.S. laboratories voluntarily submit data through the National Respiratory and Enteric Virus Surveillance System (NREVSS) established in the 1980s. NREVSS has since expanded to include monitoring of SARS-CoV-2, human parainfluenza viruses, human metapneumovirus, rhinovirus/enterovirus, respiratory adenoviruses, human coronaviruses (types 299E, NL63, OC43, HKU1), rotavirus, and norovirus. In addition to NREVSS, state and regional PHLs, a subset of hospital laboratories, and Department of Defense laboratories participate as WHO Collaborating Laboratories [30] and voluntarily transmit influenza test results weekly to the CDC. The first transmissions were by FAX then email, and since 1997, through the web-based FluNet portal [31], combining reporting and collecting influenza data from WHO Collaborating Laboratories and NREVSS clinical laboratories.

Since 2004, when influenza became a reportable result in NYS, all clinical laboratories have reported positive influenza tests to the NYSDOH, while the total number of specimens tested is not reported. Through the NYS Communicable Disease Electronic Surveillance System (CDESS), laboratory-confirmed influenza-positive cases are tallied, producing a large source of alternate data not currently transmitted to the CDC [32]. In February 2005, the clinical laboratory information management system (CLIMS) was custom-built for the Wadsworth Center and included the ability to electronically transmit influenza test data from the laboratory to state epidemiologists and, since 2010, directly to the CDC as a participant as both a NREVSS and WHO Collaborating Laboratory.

The WHO/NREVSS database for surveillance includes information on the total number of influenza-positive specimens and the total number of specimens tested. A 10% influenza positivity threshold was established by the CDC to determine the start of the influenza season, which approximates a national baseline of 2.2% outpatient visits due to ILI [22]. To assess situational awareness, we applied the APHL/CDC Right Size Roadmap sample size goal [22] of testing ≥137 samples per week to analyze the NYS WHO/NREVSS electronic data from 1997–1998 to 2021–2022 data retrospectively.

### 2.2. Progression of Test Methods for Diagnosis of Influenza at the New York State PHL

Historically, influenza was diagnosed solely on clinical presentation, without laboratory procedures. The first reported isolation of a human influenza virus in 1933 in England [33] initiated laboratory diagnosis and confirmation of influenza disease [34]. The in vitro culture of influenza viruses became the “gold standard” for confirmation of influenza disease for many decades. The NYS PHL Wadsworth Center cultured influenza viruses as early as 1949, isolating the virus from eggs inoculated with patient specimens during local influenza A outbreaks [35]. Hemagglutination-inhibition assays [36] have been utilized since 1950 [37] to identify the influenza virus type with type-specific antisera [38], and were supplemented with antigen detection tests such as immunofluorescent staining in 1954 [39]. After the 1918 influenza virus hemagglutinin gene sequence was made publicly available in 1999 [40], the Wadsworth Center initiated molecular diagnosis in 2003 by developing conventional multiplex RT-PCR assays and inaugurated real-time RT-PCR laboratory-developed tests (LDTs) in 2005 to detect and distinguish influenza A and B. In 2008, CDC developed a 5-target rRT-PCR assay, [41] subsequently utilized during the 2009 pandemic with emergency use authorization from the Food and Drug Administration, replacing the state LDTs. Automated nucleic acid extraction, robotic liquid handlers, extensive use of barcoding, and electronic data transfer provided significant enhancements to capacity and reduced turnaround time.

### 2.3. Novel Influenza Virus Detection with New York State PHL Data Sources

The CDC Flu rRT-PCR Dx Panel, available at PHLs, detects A and B influenza types and distinguishes subtypes A(H1N1)pdm09, A(H3N2), and lineages B/Yamagata from B/Victoria. Among specimens testing positive with the panel during routine surveillance, any early detection of a novel influenza virus initiates response efforts. For example, samples testing inconclusive or presumptive positive for an A(H3N2)variant, A(H5N1), or A(H7) subtype or testing positive in the typing assays but failing to produce a signal on the seasonal subtyping assays are shipped to CDC immediately for further investigation. To detect a novel virus, thresholds for the number of samples tested at PHLs are established for each state proportional to its population, and data are aggregated on a national level by the CDC. According to the Influenza Virologic Surveillance Right Size Roadmap sample sizes, during high season, NYS must test at least 124 samples per week to achieve the goal of detecting one novel virus among 700 positive specimens. Conversely, during low or shoulder season, NYS must test at least 36 samples per week to detect one novel virus among 200 positive specimens. Testing at the Wadsworth Center provides most of the influenza data for NYS required for detecting a novel virus. Other data sources have included the regional PHLs in New York City, Westchester County and Erie County, and out-of-state PHLs reporting test data on traveling NYS residents with influenza illness.

Only the CDC Flu rRT-PCR Dx Panel utilized by PHLs yields sufficient detail of influenza subtypes and lineages to detect a novel emerging strain. The Roadmap Sample Size Goals were used retrospectively to analyze the electronic NYS PHL data from 1997–1998 to 2021–2022. For both situational awareness and novel virus detection, the recommended sample sizes are based on the 2020 US Census of NYS with a total population of 20,201,249; variations in population estimates from 1997 to the present do not alter the recommended sample sizes.

## 3. Results

### 3.1. Situational Awareness for Influenza Surveillance in New York State

Influenza-like illness activity levels are considered to be elevated when percentages of cases exceed the baseline levels [30]. The baseline is established by averaging the percentage of outpatient visits for ILI symptoms in the previous three seasons during weeks when little or no influenza virus was circulating in a region. For our analysis of NYS data, peak influenza activity has been considered as 5% ILI, and ≥20% of specimens testing positive, with a 95% confidence level [32].

**Figure 2:**
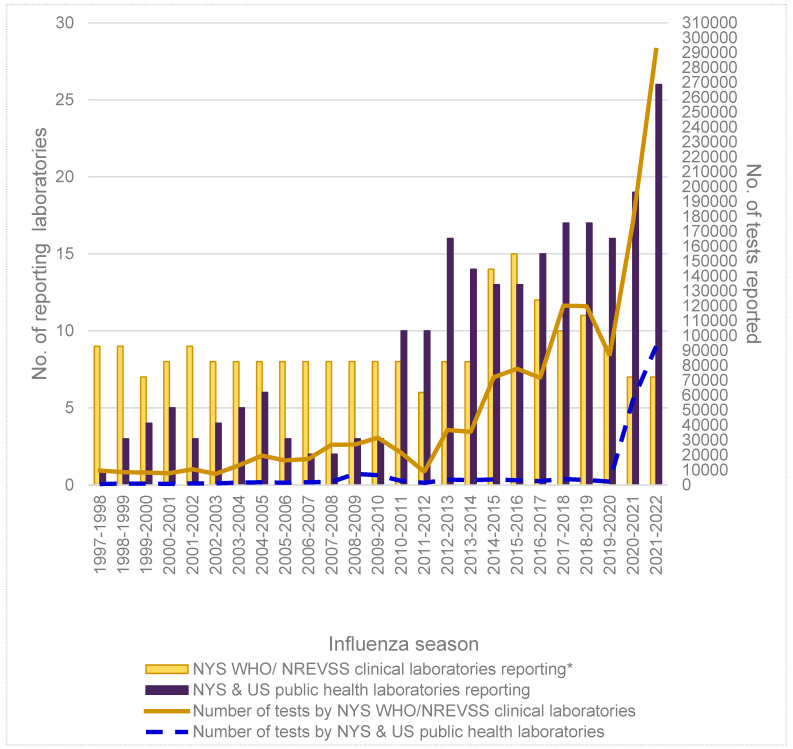
The number of public health laboratories and World Health Organization/National Respiratory and Enteric Virus Surveillance System (WHO/NREVSS) clinical laboratories* reporting the number of New York State (NYS) influenza tests to US Centers for Disease Control and Prevention for the 1997–1998 to 2021–2022 seasons. * Since 2014–2015, one WHO/NREVSS clinical laboratory outside NYS has been reporting.

From the 1997–1998 to the 2021–2022 seasons, the number of laboratories reporting influenza test results for NYS residents has grown significantly, particularly after the 2009 pandemic (Figure 2).

**Figure 3:**
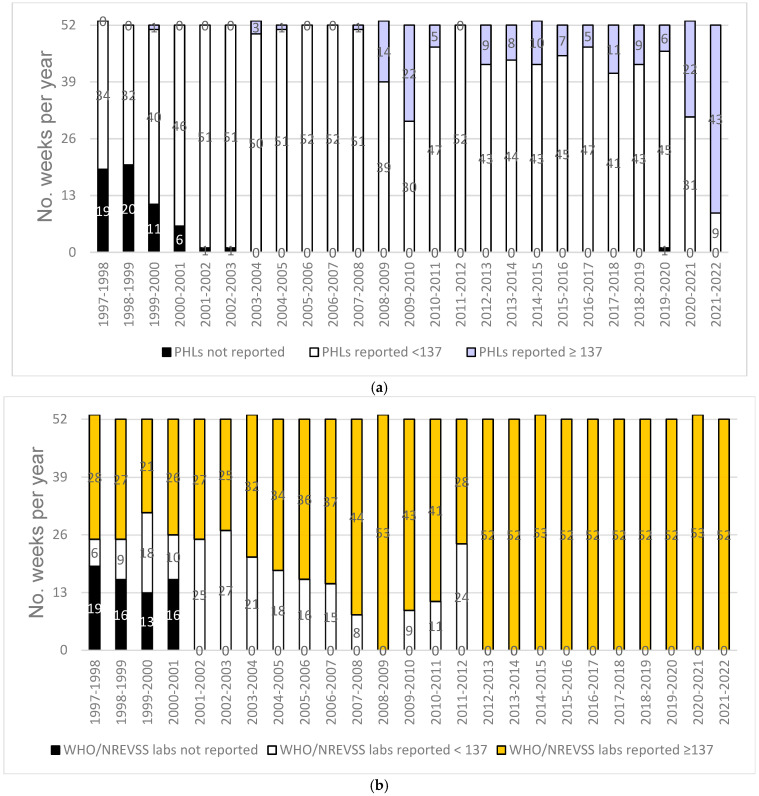
The number of weeks the New York State (**a**) public health laboratories (PHLs) and (**b**) World Health Organization/National Respiratory and Enteric Virus Surveillance System (WHO/ NREVSS) clinical laboratories reported positive results that met the ≥137 sample size goal for situational awareness according to the Influenza Virologic Surveillance Right Size Roadmap for the 1997–1998 to 2021–2022 seasons.

The greater number of laboratories reporting also yields greater numbers of specimens tested and reported. Interestingly, the total number of specimens tested during the 2009 pandemic was less than during more recent seasons. The number of laboratories reporting results also correlates with an increase in the total number of weeks that data were reported for each influenza season. While reporting increased from NYS clinical laboratories, NYS PHLs have never received sufficient sample numbers to meet the Roadmap recommended sample size goal of testing ≥137 per week for surveillance throughout an entire season (Figure 3a). However, WHO Collaborating Laboratories in NYS surpassed this recommended minimum sample size for all 52 weeks, first during the 2009 pandemic and since 2012–2013 when the Right Size Roadmap was released (Figure 3b).

### 3.2. Progression of Diagnostics for Detection of Influenza Infection

**Figure 4:**
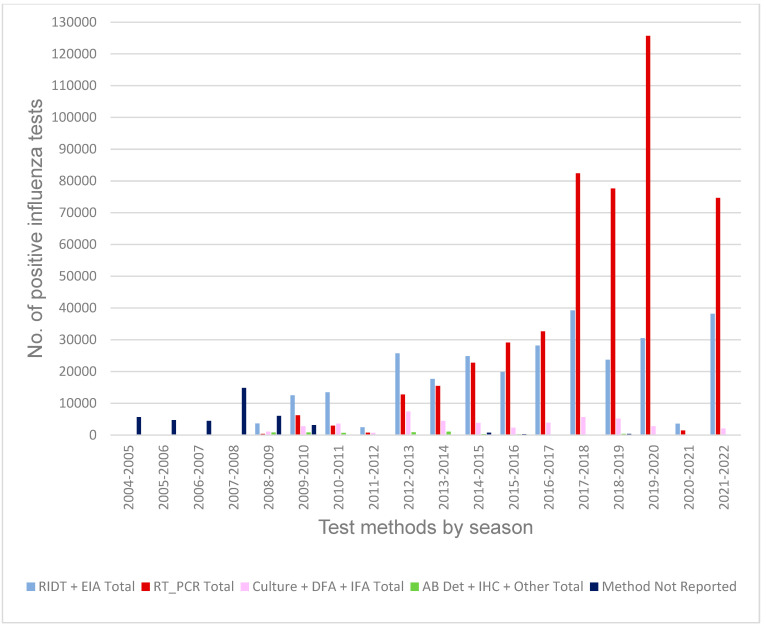
Test methods used for laboratory confirmation of influenza cases in New York State for the 2008–2009 to 2021–2022 seasons.

While data collection of influenza test methods was possible earlier, tracking of laboratory test methods correlating with detection of influenza improved markedly during the 2008–2009 season and 2009 pandemic (Figure 4).

**Figure 5:**
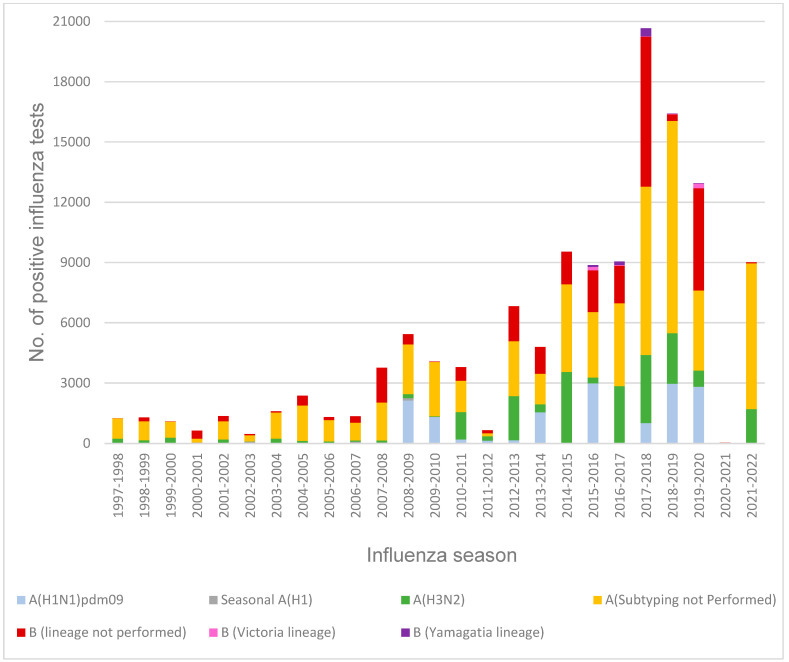
New York State positive influenza tests by type, subtype, and lineage reported by World Health Organization/National Respiratory and Enteric Virus Surveillance System (WHO/ NREVSS) clinical laboratories for the 1997–1998 to 2021–2022 seasons.

Test methods then utilized by the clinical laboratories were predominantly the rapid antigen detection assays, including the rapid influenza diagnostic tests (RIDTs) and enzyme immunoassays (EIAs) through 2014–2015. The “gold standard” of influenza virus culture confirmed by immunofluorescence assay (IFA and DFA) involves high-complexity procedures available at a decreasing number of NYS laboratories [32]. The utilization of molecular RT-PCR tests increased during the 2009 pandemic, surpassed the RIDTs in 2015–2016, doubled the number of RIDTs during the severe 2017–2018 season, and further increased with the coronavirus pandemic in 2020. These RT-PCR spikes may reflect the FDA approval of the rapid molecular platforms in recent years. This progressive development of technologies has led to increased detail in the data reported by clinical laboratories, capturing the influenza type and A subtype (Figure 5), which further enables the detection of emerging strains.

### 3.3. New York State Sample Size Goals for Novel Virus Detection

For the first eleven years of data analyzed in this article, NYS PHLs did not achieve the right size “high season” goal of testing ≥124 positive samples in one week. The peak thresholds were first met during the 2009 influenza pandemic and in about half of the following seasons. The “low season” goal of testing ≥36 and <124 positive samples per week was obtained for 19 out of 25 years. The “off-season” goal of testing one positive influenza sample in 5 weeks has been achieved every year from 1997–1998 to 2021–2022 (Table 1).

## 4. Discussion

Following the 2009 influenza pandemic, when the crisis of global recession collided with the emergence of a novel influenza strain, concerns were raised that influenza surveillance in the US was inconsistent across the country in terms of methods and quality. Further, test numbers in many states were insufficient to guarantee detection, with any reasonable level of confidence, of a novel new strain with pandemic potential at an early stage of its emergence. A joint effort by the APHL and CDC addressed this with a major initiative, culminating in the Right Size project and the generation of the Influenza Virologic Surveillance Right Size Roadmap. These provided detailed guidance on methods and test numbers for varying population sizes that would result in appropriate and sufficient data to assess situational awareness and the presence of novel strains with reasonable confidence. In this paper, these methods and testing levels were assessed in NYS, and applied to historical electronic databases of influenza test records, and we demonstrate the progress made in influenza surveillance in this state from the 1918 influenza pandemic to the COVID-19 pandemic.

State, national, and global influenza surveillance has been enhanced by the establishment of networks by WHO and CDC for data collection, which enables clinical and PHLs to report through WHO Collaborating Laboratory and NREVSS networks. NYS PHLs also participate in additional surveillance systems for CDC, including the Epidemiology and Laboratory Capacity for Infectious Diseases (ELC) [42], ILINet, and the Emerging Infections Program (EIP) [30], all of which include NYS as a participating state. Influenza-associated pediatric deaths and antiviral resistance are also reported to the CDC, and the geographic spread of influenza activity is tracked through the State and Territorial Epidemiologists Reports. ILI-Syndromic Surveillance data from emergency departments is reported to the CDC through the National Syndromic Surveillance Program, while pneumonia and influenza mortality data are collected by the National Center for Health Statistics Mortality Surveillance System [43].

Surveillance was strengthened further through the development of the Influenza Virologic Right-Size Roadmap, released by APHL and CDC in 2013 and updated in 2022 [22]. Situational awareness monitors the distribution and activity of disease throughout the year. Detection of a novel virus among positive specimens tested at state PHLs relies on the aggregation of state data for national surveillance. If the presence of a novel influenza virus is confirmed, state and federal public authorities direct an investigation into the prevalence of this virus with targeted surveillance, as in 2012 following the detection of the influenza H3N2 variant virus at state and county fairs [44]. The severe 2017–2018 influenza season generated an unprecedented number of ILI visits, boosted by extensive media coverage and heightened awareness. The total number of specimens tested for influenza almost doubled that of any previous year, including the 2009 pandemic, and the number of positive samples detected thereby increased. Interestingly, this record number of influenza tests achieved the same Right Size Roadmap thresholds as the 2009 pandemic. Testing decreased slightly in 2018–2019 and more so in 2019–2020 when efforts for influenza surveillance were diverted to the coronavirus pandemic response. During 2020–2021, the submission of specimens surged, and testing nearly doubled the peak two years prior, most likely due to the development of more respiratory panels. However, the coronavirus pandemic mitigation measures also impacted the spread of influenza: despite the record number of tests, the number of influenza-positive samples detected flatlined in 2020–2021 [45]. While the coronavirus pandemic was paramount, the Right Size calculators were applied to SARS-CoV-2 sequencing efforts, and the Roadmap strategies may be evaluated for future surveillance of other respiratory pathogens [22].

In recent years, most clinical laboratories have shifted from rapid antigen assays and high-complexity molecular assays to rapid molecular assays and molecular respiratory panels. The development of culture-independent diagnostic tests (CIDTs), both antigen-based and molecular, has led to improvements over culture in speed to result and throughput, and for molecular tests, also increased sensitivity [46]. Additionally, communication technologies enable the electronic transmission of test results in real-time for analysis by health agencies. One consequence of these advancements, however, is that fewer clinical laboratories send specimens to PHLs for influenza testing or characterization. This reduced sample submission was exacerbated by the widespread use of at-home rapid antigen tests during the COVID-19 pandemic, which has evolved into additional at-home tests for influenza. However, specimens collected and tested at home are routinely discarded. To ensure sufficient surveillance, specimen submissions to PHLs from clinical laboratories and physicians’ offices are needed because only molecular tests at PHLs identify novel subtypes and resistant strains. It is evident that NYS PHL data provide insufficient test numbers for situational awareness compared to the volume of specimens tested at WHO Collaborating and NREVSS Laboratories.

The granularity of data has increased as molecular detection methodologies evolved and now includes influenza virus type, subtype, and lineage. The Wadsworth Center tests respiratory specimens with the CDC Flu rRT-PCR Dx Panel, and results are compiled with all US PHLs into a national database by the CDC, thereby meeting the national goals of detecting a novel virus. However, NYS needs to ensure that sufficient specimen numbers are tested relative to the NYS population, and these numbers have been chronically below the recommended sample size goal. Maintaining or increasing the submission of specimens to the state PHLs remains crucial. The Wadsworth Center continues to culture viruses from respiratory specimens, a technique still critical for providing viruses for specialized biological testing and influenza vaccine production. Since 2009, the Wadsworth Center Virology Laboratory has performed pyrosequencing for drug resistance markers on influenza samples sent from other state PHLs that do not have this capability. Results of this testing are transmitted to the CDC for monitoring resistance and susceptibility to antiviral therapies. The Wadsworth Center also monitors for antiviral resistance and antigenic characterization through next-generation sequencing (NGS), creating a data set that can also be used to identify clades and subclades. NGS has greatly added to the arsenal of tools for improved diagnosis and surveillance. NYS is currently one of three National Influenza Reference Centers in the USA [47], performing NGS on specimens submitted from PHLs in 20 states and territories and assisting in the provision of data for advancing technologies and vaccine candidate selection, as a result of the national networks established by CDC in synchrony with WHO.

Despite significant improvements in influenza prevention, surveillance, diagnostics, and treatments, the global threat of an influenza pandemic continues. The highly pathogenic avian influenza virus A(H5N1) has persisted since it was first detected in humans in 1997 [48,49]. Pandemic potential is ominous for influenza A(H7N9) [50], first identified in humans in 2013 [51]. China has since witnessed six H7N9 epidemics with a fatality rate of 39%, including the largest epidemic during the 2016–2017 influenza season [52]. Among the novel influenza A viruses, the Influenza Risk Assessment Tool (IRAT) has rated the Asian A(H7N9) virus as having the greatest potential pandemic risk of public health impact, compared to the A(H5N1) virus with a higher emergence risk [53]. With extensive antigenic and genetic diversity inherent among influenza virus surface proteins, a strain to which humans are immunologically naïve could jump the species barrier at any time. Avian A(H5N1) and A(H7N9) influenza viruses are two such examples. However, swine, recognized as a “mixing vessel” for influenza viruses, have been increasingly demonstrated as a source of zoonotic influenza infections [54,55]. Recent outbreaks of highly pathogenic avian influenza A(H5N1) in dairy cows, cats, and numerous mammals, and infections of farm workers raise concern for the potential risk of transmission from multiple sources, including consumption of raw milk [56].

The Wadsworth Center NYS PHL has increased vigilance in addressing the threat of avian influenza since the virus was first detected in wild birds in 2022. The Wadsworth Center has partnered with the local health department colleagues to monitor the health of residents exposed to birds and cattle infected with the H5N1 virus. Informational messaging was disseminated to educate farm workers and operators on how to protect their health, recognize the symptoms of avian influenza infection, and seek testing should illness be suspected. Also, plans are established on how to best collect and transport any clinical specimens and initiate antiviral prophylaxis and treatment for exposed and ill individuals as appropriate. Additionally, to enhance influenza surveillance, the NYSDOH sent multiple advisories to NYS providers, requesting additional submissions to the Wadsworth Center for testing and subtyping. Further, the extensive NYS Wastewater Program, initiated in March 2020 for COVID-19, developed and validated assays for influenza detection and subtyping and performed pilot testing across two previous respiratory seasons from 2022 to 2024. Those tests, now also multiplexed with testing for respiratory syncytial virus, were implemented for testing of wastewater across NYS in November 2024. Under the federal Public Health Emergency Preparedness program, the NYS Office of Health Emergency Preparedness has worked with the local health departments, the NYS Office of Emergency Management, and other state and community partners. These efforts and the One Health concept, which strives for worldwide interdisciplinary collaboration and communication regarding all facets of healthcare for humans, animals, and the environment [57], have prompted a strong call for increased surveillance at the human–animal interface [58]. Although achievements in influenza detection, surveillance, and treatment have been substantial and enhanced further by developments in responding to the coronavirus pandemic, public health sectors are still insufficiently prepared for the next influenza pandemic [59].

## Figures and Tables

**Table 1:** New York State (NYS) public health laboratory (PHL) progress in meeting the sample size goals for detecting novel influenza viruses for the 1997–1998 to 2021–2022 seasons ^†^.

Influenza activity	High	Low	Off season
Detection thresholds	1/700	1/200	1/4
NYS PHL sample size goal	124	36	1
# weeks NYS achieved goals per season			
1997–1998	0	0	34
1998–1999	0	1	32
1999–2000	0	2	39
2000–2001	0	0	46
2001–2002	0	1	50
2002–2003	0	0	51
2003–2004	0	4	49
2004–2005	0	2	50
2005–2006	0	0	52
2006–2007	0	0	52
2007–2008	0	5	47
2008–2009	8	7	38
2009–2010	5	4	43
2010–2011	0	11	41
2011–2012	0	2	50
2012–2013	6	4	42
2013–2014	0	15	37
2014–2015	4	9	40
2015–2016	4	7	41
2016–2017	0	14	38
2017–2018	8	7	37
2018–2019	7	9	36
2019–2020	2	10	40
2020–2021	0	0	53
2021–2022	0	4	48

^†^ Surveillance objectives for detection of a novel influenza virus (a new human reassorted or animal-origin virus) are described in the Influenza Virologic Surveillance Right Size Roadmap [22]. Confidence level ≥ 95%, margin of error ≤ 5%. The color highlighted in blue for high season, green for low season, and yellow for off-season emphasized the progression in the number of weeks that the goals were achieved from 1997 to 2022.

## Data Availability

The data that support the findings of this study are available from the corresponding author upon reasonable request.

## Abbreviations

APHL	Association of Public Health Laboratories
BCDC	NYSDOH Bureau of Communicable Disease Control
BSDS	Bureau of Surveillance and Data Systems
CDC Flu rRT-PCR Dx panel	CDC human influenza real-time reverse transcription PCR diagnostic panel
CDC	Centers for Disease Control and Prevention, formerly the Communicable Disease Center
CDESS	Communicable Disease Electronic Surveillance System
CIDTs	culture-independent diagnostic tests
CLIMS	clinical laboratory information management system
DFA	direct immunofluorescence assays
EIA	enzyme immunoassays
EIP	Emerging Infections Program
ELC	Epidemiology and Laboratory Capacity for Infectious Diseases
FluNet	global web-based portal to report influenza data to CDC and WHO
GISRS	Global Influenza Surveillance and Response System
HPAI	highly pathogenic avian influenza
IFA	immunofluorescence assays
IHC	Immunohistochemistry
ILI	influenza-like illness
ILINet	outpatient surveillance network for influenza-like illness
IRAT	Influenza Risk Assessment Tool
LDTs	laboratory-developed tests
NGS	next generation sequencing
NREVSS	National Respiratory and Enteric Virus Surveillance System
NYS	New York State
NYS PHL	New York State public health laboratory, renamed the Wadsworth Center in 1984
NYSDOH	NYS Department of Health
PAHO	Pan American Health Organization
PHL	public health laboratory
RIDTs	rapid influenza diagnostic tests
RT	reverse-transcriptase
WHO	World Health Organization
